# Investigation of α-Synuclein Amyloid Fibrils Using the Fluorescent Probe Thioflavin T

**DOI:** 10.3390/ijms19092486

**Published:** 2018-08-23

**Authors:** Anna I. Sulatskaya, Natalia P. Rodina, Maksim I. Sulatsky, Olga I. Povarova, Iuliia A. Antifeeva, Irina M. Kuznetsova, Konstantin K. Turoverov

**Affiliations:** 1 Laboratory of Structural Dynamics, Stability and Folding of Proteins, Institute of Cytology of the Russian Academy of Science, Tikhoretsky ave. 4, 194064 St. Petersburg, Russia; ansul@mail.ru (A.I.S.); natalia240994@gmail.com (N.P.R.); m_sulatsky@mail.ru (M.I.S.); olp@incras.ru (O.I.P.); julgag@yandex.ru (I.A.A.); imk@incras.ru (I.M.K.); 2 Institute of Physics, Nanotechnology and Telecommunications, Peter the Great St.-Petersburg Polytechnic University, Polytechnicheskaya 29, 195251 St. Petersburg, Russia

**Keywords:** α-synuclein, amyloid fibrils, fibrillogenesis, thioflavin T, equilibrium microdialysis, binding parameters, structural polymorphism

## Abstract

In this work, α-synuclein amyloid fibrils—the formation of which is a biomarker of Parkinson’s disease—were investigated using the fluorescent probe thioflavin T (ThT). The experimental conditions of protein fibrillogenesis were chosen so that a sufficient number of continuous measurements could be performed to characterize and analyze all stages of this process. The reproducibility of fibrillogenesis and the structure of the obtained aggregates (which is a critical point for further investigation) were proven using a wide range of physical-chemical methods. For the determination of ThT-α-synuclein amyloid fibril binding parameters, the sample and reference solutions were prepared using equilibrium microdialysis. By utilizing absorption spectroscopy of these solutions, the ThT-fibrils binding mode with a binding constant of about 10^4^ M^−1^ and stoichiometry of ThT per protein molecule of about 1:8 was observed. Fluorescence spectroscopy of the same solutions with the subsequent correction of the recorded fluorescence intensity on the primary inner filter effect allowed us to determine another mode of ThT binding to fibrils, with a binding constant of about 10^6^ M^−1^ and stoichiometry of about 1:2500. Analysis of the photophysical characteristics of the dye molecules bound to the sites of different binding modes allowed us to assume the possible localization of these sites. The obtained differences in the ThT binding parameters to the amyloid fibrils formed from α-synuclein and other amyloidogenic proteins, as well as in the photophysical characteristics of the bound dye, confirmed the hypothesis of amyloid fibril polymorphism.

## 1. Introduction

α-Synuclein is a small, soluble protein consisting of 140 amino acid residues [1,2] that is mainly synthesized in the central nervous system [3,4], but can also be found in the periphery; for example, in erythroid cells [5,6], platelets [7,8], and lymphocytes [9]. The physiological role of α-synuclein is highly diverse: it is assigned to microtubule-associated activity [10], antioxidant activity in membranes [11], has a role in neuronal plasticity [12], functions as a molecular chaperone in the formation of SNARE-complexes [13], and is involved in the functioning of the Golgi apparatus and transport vesicles [14], among others. The wide range of α-synuclein functions is in a good agreement with the fact that it is a hub protein. Hub proteins are characterized by high conformational plasticity that gives them the opportunity to change their spatial configuration depending on the environment and interact with a large number of partners. For α-synuclein, more than 30 partners were identified [15].

As with many hub proteins [16], α-synuclein is an intrinsically disordered protein that has no pronounced secondary structure in aqueous solutions [17,18]. While binding to its partners, α-synuclein can acquire significantly more compact structures [19]. One of the best characterized α-synuclein folds is the membrane or vesicle bound α-helical conformation [20,21,22,23]. Recently, a tetrameric form of α-synuclein with a stable helical structure, that is suggested to play an important role in protein homeostasis, was discovered [18,24].

It is thought that disturbance of the protein interaction with its natural partners by the influence of various endogenous and exogenous factors, including mutations, leads to the association of α-synuclein molecules with the formation of ordered aggregates and the occurrence of amyloid fibrils [18,19,25,26]. α-Synuclein fibril formation is a nucleation-dependent process in which the rate-limiting step is the spontaneous formation of small metastable oligomeric intermediates, called amyloid cores [27]. As was shown in the latest research, the initially formed proteinase K-sensitive amyloid oligomers slowly converse into more stable proteinase K-resistant oligomers [28], proceeding to protofibril formation. These protofibrils can interact with each other with the formation of highly polymorphic α-synuclein amyloid fibrils [29,30].

Aggregated α-synuclein is the major component of pathological Lewy bodies in Parkinson’s disease and dementia (DLB), as well as amyloid plaques in Alzheimer’s disease [15,27]. However, the processes underlying α-synuclein aggregation and its pathological appearance are currently not well understood. Elucidation of the molecular mechanisms of α-synuclein amyloidosis (synucleinopathys), study of the deposits’ properties, elaboration of methods of in vivo diagnosis of these pathological inclusions, and development of therapeutic treatments are urgent tasks. A successful solution of these problems depends largely on our knowledge about the physicochemical properties, structural features, and formation mechanism of α-synuclein amyloid fibrils, as well as the mechanisms of their interaction with potential diagnostic and therapeutic agents.

One of the widely used tools for the diagnosis of amyloid fibrils, and, recently, for investigation of their structure is the benzothiazole dye thioflavin T (ThT) [31,32,33,34,35]. This is due to the specificity of the dye interaction with amyloid fibrils, and the significant difference of its photophysical characteristics in the free and bound to fibrils states. Recent works have shown that determination of ThT-amyloid fibril binding parameters can be used for investigation of their structure [34,35,36]. However, there are no studies where stoichiometry and affinity of the dye interaction with α-synuclein amyloid fibrils were evaluated correctly. In most studies of ThT binding parameters for amyloid fibrils formed from α-synuclein and other proteins, the dependence of the dye fluorescence intensity on its total concentration was used instead of the concentration of the free dye [37,38,39,40]. In addition, in these studies, the influence of the inner filter effect on the detected fluorescence intensity was either not taken into account at all, or was performed incorrectly. To solve these problems, in the present work a specially elaborated approach based on spectral investigation of the solutions prepared by equilibrium microdialysis [41], and a specially developed method of inner filter effect correction [42], were used. The applicability of the proposed method for binding stoichiometry and affinity determination was earlier demonstrated in a study of the ThT interaction with amyloid fibrils formed from the different amyloidogenic proteins [41,43], and fluorescent dye 8-Anilino-1-naphthalenesulfonic acid (ANS) with serum albumins [44].

In the present work, the approaches mentioned above were used for estimation of the ThT-α-synuclein amyloid fibril binding parameters. For determination of the duration of α-synuclein fibrillogenesis in the experimental conditions, as well as to verify the reproducibility of this process and the structure of the obtained aggregates, the kinetics of fibril formation and mature aggregates were preliminarily investigated using a wide range of physical-chemical methods.

## 2. Results and Discussion

### 2.1. Kinetics of α-Synuclein Fibrillation

Analysis of the results of the previous work [45] allows us to conclude that the conditions of α-synuclein fibrillogenesis can significantly affect both the kinetics of this process and the properties of mature amyloid fibrils. The reproducibility of fibrillogenesis, and hence the structure of mature amyloid fibrils, is an important point for further investigation. The most optimal duration of fibrillogenesis to study various parameters of this process is from several hours to 24 h, since during this period a sufficient number of continuous measurements can be performed to characterize and analyze all stages of this process. However, it turned out that under physiological conditions, α-synuclein fibrillogenesis continues for more than three days [45]. At the same time, duration of this process is significantly reduced at high temperatures or acidic pH conditions. Taking into account the abovementioned time limits, α-synuclein fibrillogenesis was performed at pH 3.7 in 0.2 M acetate buffer with constant stirring at 37 °C.

**Figure 1:**
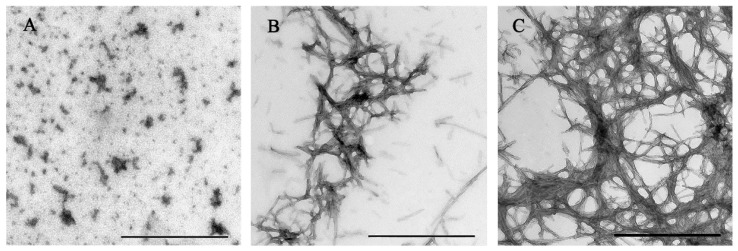
Electron micrographs of α-synuclein during protein aggregation: (**A**) At the lag-phase (when the ThT fluorescence intensity and the CD spectrum of the protein do not change); (**B**) at the growth phase (when ThT fluorescence intensity increases and the shape of the CD spectrum changes significantly); and (**C**) at the phase of mature amyloid fibrils (when the dependence of the ThT fluorescence intensity on time reaches a plateau and the CD spectrum ceases to change). Scale bar is 1 μm.

**Figure 2:**
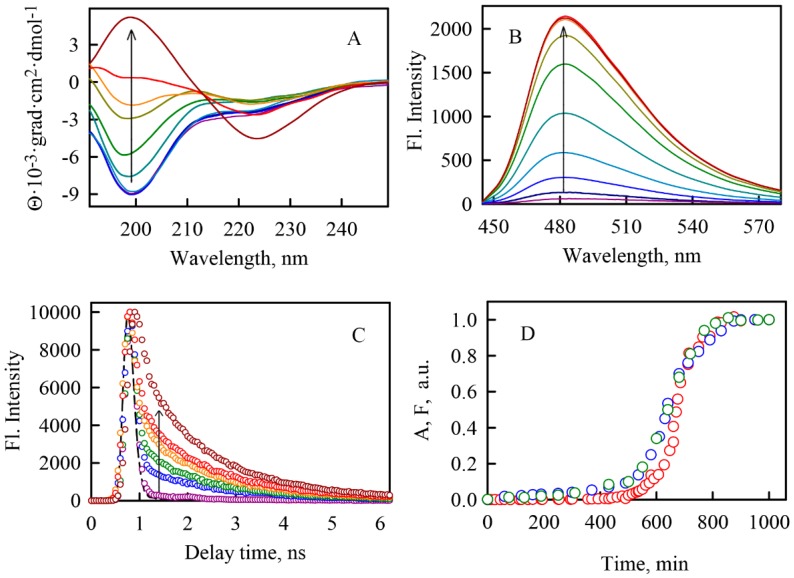
Time-dependent change of the photophysical characteristics of α-synuclein and the fluorescent probe thioflavin T (ThT) during fibrillogenesis. Arrows indicate the direction of change. (**A**) The CD spectra of α-synuclein at 0, 190, 310, 430, 670, 710, 790, 830, 880, and 1000 min; (**B**) ThT fluorescence spectra at 510, 560, 610, 650, 670, 710, 790, 880, 950, and 1000 min; (**C**) ThT fluorescence decay curves at 480, 610, 650, 710, 850, and 1000 min after the start of the fibrillogenesis. The instrument response function is presented by the black dashed line. Panel (**D**) shows the time dependencies of fluorescence (red circles), fluorescence excitation (blue circles), and absorption (green circles) at the maxima of the corresponding spectra.

α-Synuclein aggregates were visualized by electron microscopy (Figure 1), and changes in the protein secondary structure during protein fibrillogenesis were monitored by CD-spectroscopy (Figure 2A). The far-UV CD spectrum of the monomeric α-synuclein is typical of a predominantly unfolded protein, and is proven by a strong negative band around 200 nm [46]. Nevertheless, a weak negative band, appearing as a shoulder around 220 nm, allows us to suggest the presence of secondary structure elements in the monomeric protein. Upon fibrillogenesis, α-synuclein goes from a conformation that is predominantly unfolded to a conformation with considerable β-structure, as revealed by the concomitant intensity decrease of the 200 nm band and increase of the 220 nm band. CD spectra of proteins and peptides with representative secondary structures that can be used for the visual qualitative evaluation of the obtained results are presented, for example, in the work by Greenfield [47].

The kinetics of amyloid fibril formation is widely monitored by the fluorescence enhancement of the specific probe thioflavin T (ThT) (Figure 2B) (see, for example, Ban research [48]). In this work, we studied this process in addition to the registration of other ThT photophysical characteristics, such as fluorescence decay curves (Figure 2C), and absorption and fluorescence excitation (Figure 2D). Figure 2D shows the same sigmoid character of the time-dependent increase of the different detected parameters. The kinetic curves can be divided into three regions: the first (lag phase) is characterized by the ThT parameters’ invariability, the second is characterized by their increase, and the third is characterized by their plateau.

We compare the data obtained by CD spectroscopy and fluorescence spectroscopy in the presence of ThT with the existing models of amyloid fibril formation to conclude with which of them our data are more consistent. Our results show the CD spectrum of the protein does not change its shape up to 430 min after the beginning of the experiment, which corresponds to the lag phase (the duration of which is determined in Figure 2D using fluorescence spectroscopy in the presence of ThT). Further, during the growth phase the protein structure changes, which is confirmed by a significant change in its CD spectrum from −8400 to 5200 grad·cm^2^·dmol^−1^ at 200 nm wavelength (the peak characteristic for predominantly disordered proteins), and from −3600 to −4000 grad·cm^2^·dmol^−1^ at a wavelength of 220 nm (peak characteristic for proteins enriched with β-structure). Analysis of the literature shows that our results do not agree with the existing idea that in the lag phase all the protein passes into the form of ring oligomers and the elongation of amyloid fibrils in the growth phase occurs due to interaction of the protein ring-like oligomers formed in the lag phase [49].

**Scheme 1:**
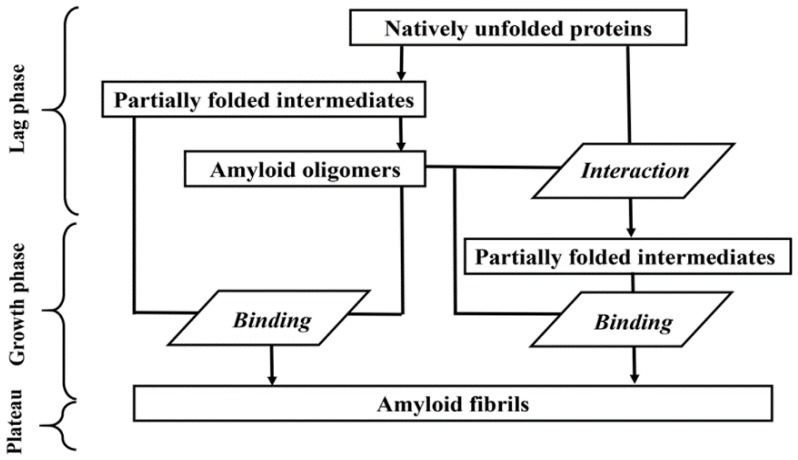
Scheme of α-synuclein fibrillogenesis. Created on the basis of the results of Uversky’s work [45].

In this fibrillogenesis scheme, the main protein structural transformations should occur in the lag phase (when the ring oligomers are formed by the main part of the protein), which should be reflected in a significant change in the CD spectra in the lag phase. However, such changes were not observed. At the same time, our results do not contradict the model presented in a previous paper [45], where the scheme of fibrillogenesis of α-synuclein is described (Scheme 1). According to this scheme, in the lag phase nucleation cores are formed, then in the growth phase the seeds (formed in the lag phase) are elongated by binding of monomeric protein molecules to the core of the growing fibril, after which the phase of mature fibrils begins.

We hypothesized that the absence of changes in the CD spectrum in the lag-phase of α-synuclein fibrillogenesis is due to the fact that only a small part of the protein participates in the formation of amyloid oligomers, while the bulk of the α-synuclein remains in monomeric form. This may be due to the fact that spontaneous formation of such oligomers is a long and low-probability process. Thus, it can be assumed that an insignificant change in the CD spectra in the lag-phase is due to the insignificant contribution of a small number of molecules that change their structure during the formation of the nucleus into the total spectrum of the sample CD. At the same time, the formed nucleation core can further act as a “matrix” for structural changes of other α-synuclein molecules (in particular, as it was previously shown for prions [50]). Based on the results of the work by Zahn [50], it can be assumed that in the growth phase proteins with a partially-folded structure (which is characteristic for the protein under the experimental conditions, according to Uversky [45]), interact with to amyloid oligomers, which leads to a change in the secondary structure of monomer proteins and an increase in the content of beta-sheets.

The obtained results show that the kinetic dependence reaches a plateau that is caused by saturation of the ThT binding sites and the formation of mature amyloid fibrils in about 15 h. For the amyloid fibril preparation for the following experiments, α-synuclein was incubated under the chosen conditions for at least 24 h.

### 2.2. ThT-α-Synuclein Amyloid Fibril Binding Parameters

Since the fluorescence quantum yield of free ThT in aqueous solutions is very low (~0.0001 [51]), and because when the dye binds to fibrils this value may increase 1000-fold, it would seem that to evaluate ThT-α-synuclein amyloid fibril binding parameters, one can use the dependence of the fluorescence intensity of ThT in the presence of amyloid fibrils (*F*) on its concentration. However, it should be noted that the solution of ThT with fibrils is an equilibrium system of free (*C_f_*) and bound to fibrils (*C_b_*) dye. In all studies on the characterization of ThT binding to amyloid fibrils for determination of ThT-fibril binding parameters, the total concentration of ThT (*C_0_*) was used instead of the concentration of the free dye (*C_f_*), which would have been preferable (for details see Supplementary Materials). Determination of *C_f_* and *C_b_*, at first sight, seems to be a very difficult task. However, to solve this problem, we proposed a simple but very effective approach that was developed a long time ago for the study of receptor–ligand interactions, but that was lately undeservedly forgotten [52]. The essence of this approach is the preparation of investigated solutions by the equilibrium microdialysis technique.

**Figure 3:**
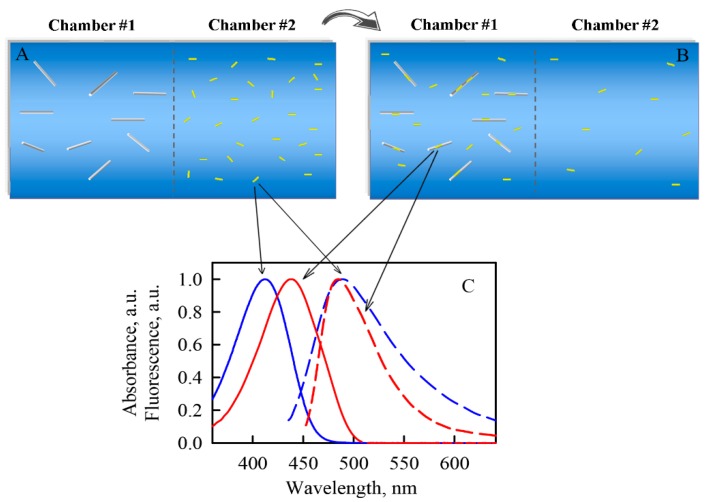
Determination of photophysical properties of ThT bound to α-synuclein amyloid fibrils by the preparation of the sample and reference solutions using equilibrium microdialysis. (**A**) α-Synuclein amyloid fibrils in the buffer solution were placed in chamber #1 and the ThT solution in the same buffer was placed in chamber #2. (**B**) After equilibration, free ThT concentrations in chambers #1 and #2 become equal, while the total ThT concentration in chamber #1 is greater than that in chamber #2 by the concentration of the bound dye. (**C**) Normalized absorption (solid curves) and fluorescence (dashed curves) spectra of the free dye in buffer solution (blue curves), and ThT bound to α-synuclein amyloid fibrils (red curves), obtained by the spectroscopic investigation of the solutions after equilibrium microdialysis.

The device for equilibrium microdialysis execution consists of two chambers (#1 and #2) divided by a membrane permeable to ThT and impermeable to amyloid fibrils. In equilibrium microdialysis experiments, mature α-synuclein amyloid fibrils (prepared under the chosen conditions for at least 24 h) were used. The procedure of the sample preparation is shown in Figure 3A. The initial concentration of ThT (*C_o_*) was changed in the range of 3 to 100 mM, and the concentration of fibrils in terms of amyloidogenic protein concentration *C_p_* was equal to 30 mM in every experiment. In equilibrium free ThT, the concentration in chamber #1 was equal to that in chamber #2 (*C_f_*), while the total ThT concentration in chamber #1 was greater than that in chamber #2 by the *C_b_* concentration (*C_f_* + *C_b_*) (Figure 3B). For determination of *C_f_* and *C_b_* values, absorption spectra of the sample solution containing free and bound to fibrils ThT, and the reference solution containing only free ThT (in a concentration equal to the concentration of free dye in the sample solution) were recorded. For the solutions containing amyloid fibrils, the contribution of the light scattering was taken into account.

**Figure 4:**
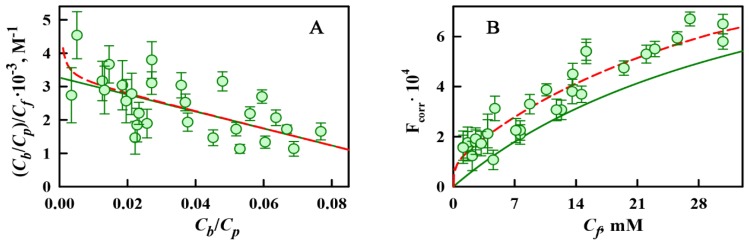
Determination of ThT-α-synuclein amyloid fibril binding parameters. (**A**) Scatchard plot and (**B**) dependence of the fluorescence intensity (corrected on the primary inner filter effect) on the concentration of free ThT are presented. Experimental data (circles) and best-fit curves calculated with the use of the binding constants (*K_bi_*) and number of binding sites (*n_i_*), calculated according to the assumption of one (green solid curves) and two (red dashed curves) binding modes existing, are given. The model of two binding types of ThT is discussed in Section 2.3.

To get a visual representation of the number of ThT-α-synuclein fibril binding modes, the Scatchard dependence using the calculated *C_f_*, *C_b_*, and *C_p_* (concentration of the protein on the basis of which the amyloid fibrils were prepared) was plotted (Figure 4A). The linearity of this dependence indicates the identity of all binding sites; that is, the existence of one ThT-α-synuclein amyloid fibril binding mode (hereinafter referred to as the “first” binding mode). Thus, the value of the binding (association) constant (*K_bi_*) and the number of binding sites (*n_i_*) were determined by the assumption that the number of binding modes *i* = 1, using the equation:

(1) $$C_{b}=\sum_{i}\frac{n_{i}C_{p}C_{f}K_{bi}}{1+C_{f}K_{bi}}$$

The obtained values of the binding parameters of ThT to α-synuclein amyloid fibrils, as well as to amyloid fibrils formed from other amyloidogenic proteins [41], are presented in Table 1. It should be noted that the ThT-α-synuclein fibril binding constant has the same order of magnitude as the binding constant of the dye to one of the modes of other amyloid fibrils, especially those formed from insulin, lysozyme, and Aβ-peptide. This fact may indicate the similarity of the mechanism of the dye interaction with these amyloid fibrils. We believe that this type of interaction is due to the incorporation of ThT molecules into the grooves formed by the side chains of amino acides, along the long axis of the fibril perpendicular to the β-sheets [53]. However, observed differences in the value of the binding affinity and stoichiometry suggest differences in the structure of this binding site, and consequently in the structure of amyloid fibrils formed from various proteins.

### 2.3. Photophysical Characteristics of ThT Bound to α-Synuclein Amyloid Fibrilsα

Spectroscopic investigation of the solutions prepared by equilibrium microdialysis allowed us to determine not only the ThT-amyloid fibril binding parameters, but also the characteristics of the bound dye. Using the proposed approach, the absorption spectra of the ThT bound to α-synuclein amyloid fibrils were determined for the first time (Figure 3C). As in the case of amyloid fibrils on the basis of other amyloidogenic proteins [41,43], the absorption spectra of ThT incorporated into α-synuclein amyloid fibrils are long-wavelength shifted. In order to understand the causes of the observed effects, it is necessary to pay attention to the fact that ThT is a cation, and the positive charge of the dye molecule is distributed unevenly between its fragments. ThT can be considered as a molecule with a uniformly distributed charge, and a dipole with positive and negative charges concentrated on the dye benzothiazole and aminobenzene rings [54]. ThT molecules in the ground state in polar solvents are stabilized by the orientational interaction of the polar dipoles of the solvent with the dye dipole. When the transition to an excited state occurs, the charge distribution between the ThT fragments significantly changes. This leads to the configuration of the solvation shell of the ThT molecule in the excited Frank–Condon state becoming becomes nonequilibrium with the solvent. Thus, in polar solvents, the light absorption by the ThT molecule is due to the transition from the equilibrium ground state to the nonequilibrium excited state. Thereby, the more polar the solvent is, the shorter the wavelength position of the absorption spectrum that can be observed for the free dye in the aqueous solution. When the dye bonds to amyloid fibrils, its microenvironment changes (becomes more hydrophobic), which leads to a noticeable shift of the absorption spectrum to the long-wavelength region.

The determined absorption (*A_b_*) and concentration (*C_b_*) of ThT bound to α-synuclein amyloid fibrils allowed us to calculate the molar extinction coefficient of the bound dye for the first time. It was shown that when the dye binds to fibrils, along with the shift of the ThT absorption spectra, a decrease in its molar extinction coefficient can be observed (Table 1). At the same time, for amyloid fibrils formed from other amyloidogenic proteins an increase of this value was shown. It can be assumed that these differences are caused by the various structures of the amyloid fibril binding sites, which in turn leads to differences in the bound ThT molecules’ microenvironment and conformation.

In the next step, prepared solutions were investigated by fluorescence spectroscopy. Using the sample and reference solutions obtained by equilibrium microdialysis, the fluorescence spectra of ThT bound to amyloid fibrils were determined. The position of the fluorescence spectrum of ThT bound to amyloid fibrils coincides with the position of the spectrum of the monomer dye in aqueous solution (Figure 3C). This fact confirms the monomeric form of the dye binding to amyloid fibrils [55]. Weakly depending the position the fluorescence spectrum of the dye on the polarity of the solvent was first shown in the paper by Maskevich [54]. This is due to the fact that fluorescence (in contrast to absorption) is determined by the transition of a dye molecule from a partially-equilibrium excited state to a partially-equilibrium ground state.

The recorded values of fluorescence intensity were corrected on the primary inner filter effect with the use of a specially developed approach (for details see Supplemenary Materials) [42]. This allowed us to determine the fluorescence quantum yield of ThT bound to α-synuclein amyloid fibrils (*q_i_*) (see Table 1). It can be noted that its value is two orders of magnitude greater than the fluorescence quantum yield of the free dye in aqueous solution. The increase of the fluorescence quantum yield of ThT bound to amyloid fibrils is caused by the molecular rotor nature of the dye. Rotation of ThT benzothiazole and aminobenzene rings relative to each other in the excited state leads to the dye conformation with the disturbed π-electron conjugated system (*φ* ≈ 90°) of these fragments and, consequently, to the radiation-less deactivation of the excited state of free ThT molecules in solution [54]. The fluorescent quantum yield increase can be caused by restriction of the ThT fragments’ rotation relative to each other during the dye incorporation into the “grooves” of amyloid fibrils.

**Figure 5:**
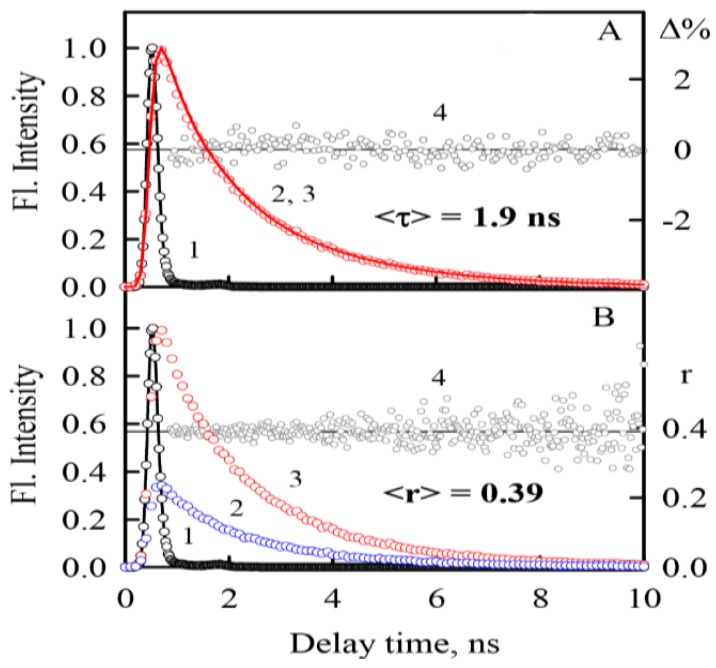
Time characteristics of fluorescence of ThT bound to α-synuclein amyloid fibrils. Panel (**A**) shows the instrument response function (1), experimental fluorescence intensity decay curve of the bound dye (2), best fit calculated fluorescence decay curve (3), deviation between the experimental and calculated decay curves (4), and the value of the average lifetime of the excitation state calculated on the presented experimental data. Panel (**B**) shows the instrument response function (1), the decay curves of the vertical (2) and horizontal (3) components of fluorescence intensity, the time dependence of the fluorescence anisotropy (4), and the value of the average fluorescence anisotropy.

It was also shown that, when ThT interacts with α-synuclein amyloid fibrils, its florescence lifetime increases significantly (from 0.001 ns for ThT in water solution [56] to 1.9 ns for bound to fibrils dye) (Figure 5A). ThT’s fluorescence lifetime in aqueous solutions is determined by the rate of internal rotation of its fragments relative to one another, because the rate of this process is much higher than the rotation of a whole ThT molecule. The observed increase of the dye florescence lifetime value when it binds to amyloid fibrils can be explained by the restriction of the rotational motions of ThT benzothiazole and aminobenzene rings in the excited state, and the decrease of the radiation-less deactivation rate of the dye molecules.

At the same time, it was shown that fluorescence anisotropy of ThT incorporated into α-synuclein amyloid fibrils remains unchanged (Figure 5B). This is due to the fact that the dye anisotropy in aqueous solutions is about 0.38 [56], which is close to the upper limiting value. This fact is caused by the molecular rotor nature of ThT. Rotation of the whole ThT molecule (which leads to the dye fluorescence anisotropy change) is two orders of magnitude slower than the dye fragments’ internal rotation relative to one another. At the same time, the axis of the dye fragments’ rotation coincides with direction of the transition dipole moment of ThT, and consequently cannot change direction but leads to radiation-less deactivation of the dye molecules and a short fluorescence lifetime. Therefore, during the fluorescence lifetime the dye molecule does not have enough time to change its spatial orientation, leading to high ThT fluorescence anisotropy in aqueous solutions. When the dye bound to fibrils ThT fluorescence anisotropy remains high, it is because the characteristic time increases simultaneously for the both these process (rotations).

### 2.4. The Possibility of the Existence of One More ThT-α-Synuclein Amyloid Fibril Binding Mode

**Figure 6:**
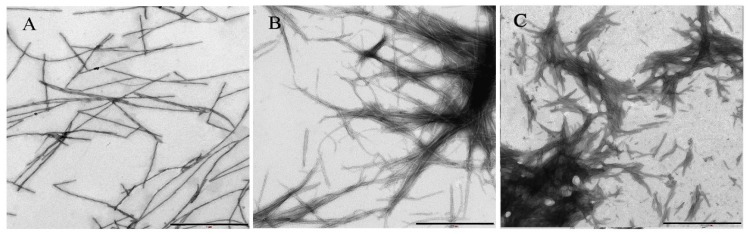
Electron micrographs of (**A**) β-2-microglobulin, (**B**) insulin, and (**C**) lysozyme amyloid fibrils. Scale bar is 1 μm.

**Figure 7:**
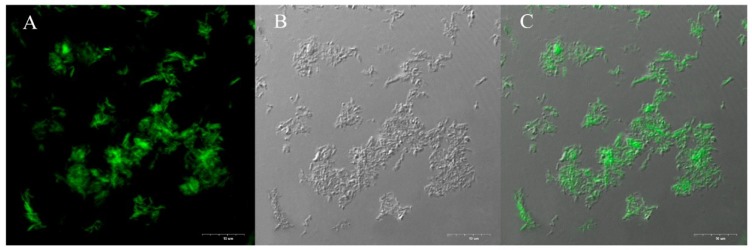
Confocal microscopy images of α-synuclein amyloid fibrils. (**A**) Fluorescence image of the ThT-stained fibrillar structures, (**B**) transmitted light image, and (**C**) overlay of these images. Scale bar is 10 μm.

Along with the binding mode with the affinity ~10^4^ M^−1^ in amyloid fibrils on the basis of various proteins, another binding mode with higher affinity ~10^6^ M^−1^ (hereinafter referred to as the «second» binding mode) can be observed (Table 1) [41]. In order to understand the nature of the second binding mode, we analyzed electron micrographs and data regarding the number of binding modes obtained earlier for various amyloid fibrils [41,57,58]. It can be noted that for amyloid fibrils formed from insulin and lysozyme (Figure 6), which tend to form aggregates, the existence of a second binding mode with high affinity was reliably confirmed using the data of absorption spectroscopy of solutions prepared by equilibrium microdialysis [41]. At the same time, it was shown that fibrils formed from the same protein (lysozyme) obtained under different conditions have a different degree of clasterization, which correlates with the number of binding sites in the second mode [58]. It was also shown that amyloid fibrils formed from various forms of β-2-microglobulin, representing an only/primarily fibrillar structure (Figure 6), have only one mode of dye binding [57]. It was suggested that the existence of the second binding mode is caused by localization of the ThT molecules in the clustered areas of the fibrils (possibly within the above-discussed grooves along the fiber axis). This localization may determine the higher binding affinity and more rigid restriction of dye fragments’ rotation relative to one another, and lead to a more significant increase of ThT fluorescence quantum yield in comparison to the case of the first binding mode. Electron microscopy data showed that α-synuclein amyloid fibrils have clustered areas as well (Figure 1), however there are less of them and they are much smaller in comparison to those of insulin and lysozyme fibrils, in which the binding mode with high affinity was found. Furthermore, confocal microscopy data that show different fluorescence intensities intensity of ThT bound to the different areas of α-synuclein amyloid fibrils (a more intense emission is detected in fibril clustering regions) do not contradict this assumption (Figure 7). In this regard, we can assume the possibility of the existence of the second ThT-α-synuclein amyloid fibril binding mode, which is difficult to detect by absorption spectroscopy because of a very small number of these binding sites.

We assumed that the second binding mode can be observed using the fluorescent spectroscopy results. This assumption is based on the fact that, besides absorption, fluorescence intensity depends on the fluorescence quantum yield of the sample. According to our previous results for fibrils formed from other amyloidogenic proteins, the fluorescence quantum yield of ThT bound to a high affinity mode can significantly exceed that of the dye bound to the first mode. This means that, despite the small number of binding sites of the second mode, the molecules interacting with it can have a noticeable contribution to the total fluorescence intensity. To verify this hypothesis it was checked whether the approximation of the experimental fluorescence intensity dependence on the free dye concentration by the calculated curve is satisfactory. The theoretical curve was plotted with the use of the obtained ThT-α-synuclein fibril binding parameters, under the assumption of only one binding mode (*i* = 1) existing.

(2) $$F_{corr}=\sum_{i}q_{bi}A_{bi}=\sum_{i}q_{bi}\varepsilon_{bi}lC_{bi}=\sum_{i}q_{bi}\varepsilon_{bi}l\frac{n_{i}C_{p}C_{f}K_{bi}}{1+C_{f}K_{bi}}$$

Here, *F_corr_* is the corrected primary inner filter effect fluorescence intensity; *q_b_* and *ε_b_* are the quantum yield and molar extinction coefficient of ThT bound to fibrils, respectively; and *l* is the optical pathlength. Figure 4B shows the mismatch of the experimental and calculated curves that, as assumed, can be caused by the existence of the second dye binding mode. ThT binding parameters for this mode were determined with the use of Equation (2), written with the assumption of the existence of two binding modes (*i* = 2) by multiple nonlinear regression.

When calculating ThT-amyloid fibril binding parameters, it should be taken into account that absorption spectroscopy is a direct method for this aim and provides more accurate results than fluorescence spectroscopy. Therefore, the ThT binding parameters calculated by absorption spectroscopy in the first mode were fixed during the calculation of binding parameters for the second mode. It should be noted that such a calculation is possible due to the preparation of the tested solutions by equilibrium microdialysis, since only this approach allows us to calculate the concentration of free dye (*C_f_*) in each sample. Furthermore, using the fluorescence intensity correction on the primary inner filter effect is a critical point, since an increase in the total concentration of the solution leads to a significant underestimation of the real fluorescence intensity values.

The obtained results (Table 1) show that the number of binding sites in the second mode of α–synuclein amyloid fibrils is very small, as expected. The calculated curves plotted with the use of the obtained binding parameters, under the assumption of the existence of two binding modes (*i* = 2), are shown in Figure 4B. Scatchard plots for the cases of one and two binding modes almost coincide (Figure 4A). Small differences are observed only in the region of low *C*_0_ values, for which the measurement error is sufficiently high and does not allow correct estimates to be made. At the same time, the dependencies of the fluorescence intensity on the free dye concentration in these cases differ significantly (Figure 4B). The better fit of the experimental data by the approximation obtained by the model of two binding modes was proved using statistical analysis (see Supplementary Materials). This confirms the hypothesis of a greater sensitivity of absorption spectroscopy to the parameters of lower affinity mode of ThT binding to α-synuclein amyloid fibrils and of a greater sensitivity of fluorescence spectroscopy to the parameters of higher affinity mode of ThT binding to these fibrils.

With the use of the obtained binding parameters and the results of absorption and fluorescence spectroscopy of solutions prepared by equilibrium microdialysis concentration, the molar extinction coefficient and fluorescent quantum yield of the dye bound to the second mode of amyloid fibrils were calculated (Table 1). As expected, the fluorescence quantum yield of the dye bound to the mode with a higher affinity is significantly (almost tenfold) higher than that of ThT bound to the first mode.

## 3. Materials and Methods

### 3.1. Materials

Thioflavin T (ThT) “UltraPure Grade” from AnaSpec (USA) was used without additional purification. ThT was dissolved in 2 mM Tris-HCl, 150 mM NaCl buffer (pH 7.7). Fluorescent dye ATTO-425 from ATTO-TEC (Germany) and buffer components from Sigma (USA) were used without additional purification.

### 3.2. α-Synuclein Expression and Purification

Wild type human α-synuclein was expressed in *E. coli* BL21 cells. The T7-7 plasmid, encoding for the wild type protein sequence, was used for transformation. This plasmid was a generous gift from Professor Donna Arndt-Jovin (Max Planck Institute for Biophysical Chemistry, Laboratory of Cellular Dynamics). A total of 100 mL of a preculture was inoculated to 900 mL with Lysogeny broth (LB) medium, with the addition of 1 mL of ampicillin at a concentration of 100 mg/mL. Incubation was carried out at 37 °C with agitation (400 rpm) until the absorption at wavelength 600 nm reached the value of 0.6–0.8. Expression of the recombinant protein was induced by addition of isopropyl β-D-1-thiogalactopyranoside (IPTG) (0.5 mM), and incubation was continued for an additional 4–6 h. The bacterial cells were harvested by centrifugation (Beckman JA10 rotor, 5000 rpm, 30 min, 4 °C) and the pellets were stored at −20 °C. The cell pellets were thawed with 15–20 mL lysis buffer containing 10 mM Tris-HCl, pH 8.0, 1 mM EDTA, and 1 mM protease inhibitor PMSF. The cells were lysed by sonication with a Labsonic M apparatus for 30 min. The mixture was centrifuged for 30 min at 5000 rpm, recovering the supernatant. Proteins other than α-synuclein were precipitated by boiling at 100 °C for 20 min, followed by centrifugation at 12,000 rpm for 30 min at 4 °C. The pellets containing α-synuclein were stored at −20 °C. Purification of α-synuclein was carried out with Mono-Q 10/10 column (Amersham Biosciences, Bath, UK).

### 3.3. Investigation of the Kinetics of Amyloid Fibril Formation

Solutions of α-synuclein at pH 3.7 in 0.2 M acetate buffer were stirred at 37 °C in glass vials with microstir bars. The protein concentration was 1 mg/mL. Fibril formation was monitored with thioflavin T absorption and fluorescence. The presence of fibrils was confirmed by electron microscopy. Changes in the protein secondary structure were monitored by CD-spectroscopy. The fibrillogenesis was repeated several times to verify the identity of the formed aggregates.

### 3.4. Electron Microscopy

To obtain electron micrographs, the method of negative staining with a 1% aqueous solution of uranyl acetate was used. The amyloid fibrils were placed on copper grids coated with a collodion film-substrate. Electron micrographs of the amyloid fibrils were obtained using a transmission electron microscope Libra 120 (Carl Zeiss, Jena, Germany).

### 3.5. Confocal Microscopy

For obtaining the fluorescence images of the ThT-stained fibrillar structures, the confocal laser scanning microscope Olympus FV 3000 (Olympus, Tokyo, Japan) was used. An objective lens with a 60× magnification and an aperture of NA 1.42 was used. For fluorescent probe excitation, the fixed laser line was used (405 nm). Registration of fluorescent light was carried out in the range of 420–520 nm. To assess the presence of fibrils in the investigated sample region and the areas of the dye accumulation, transmitted light images was also obtained.

### 3.6. Equilibrium Microdialysis

Equilibrium microdialysis was performed with a Harvard Apparatus/Amika (Holliston, MA, USA) device, which consists of two chambers (500 μL each), separated by a membrane (MWCO 10,000) impermeable to particles larger than 10,000 Da. In these experiments the mature amyloid fibrils were used in a concentration of 0.4 mg/mL. Before performing the experiments with amyloid fibrils, the duration of the equilibration was determined. We put ThT solution with a wide range of concentrations of *C*_0_ in chamber #1 and the solvent in chamber #2. After 10 h of dialysis, the absorption spectra of samples from chambers #1 and #2 coincided (*A*(λ)_#1_ = *A*(λ)_#2_). This means that 24 h is enough time to allow the dye to equilibrate between chambers #1 and #2. In addition, it was shown that *A*(λ)_#1_ = *A*(λ)_#2_ = *A*(λ)_0_/2, which means that the dye does not bind to the membrane or chamber walls.

### 3.7. Absorption Spectroscopy

The absorption spectra were recorded using a spectrophotometer U-3900H (Hitachi, Tokyo, Japan). The concentration of ThT and α-synuclein in the solutions was determined using molar extinction coefficients of *ε*_412_ = 31600 M^−1^cm^−1^ (on the basis of our results) and *ε*_280_ = 5120 M^−1^cm^−1^ [59], respectively. The contribution of light scattering to the measured absorption spectra was determined from the relationship: *A_sca_*_t_ = *aλ^−m^* [60]. The coefficients *a* and *m* were determined from the linear part of the dependence of *A* on *λ*, where there is no active dye absorption plotted in the logarithmical coordinates *lg*(*A_scat_*) = *f*(*lg*(λ)).

### 3.8. Fluorescence Spectroscopy

Fluorescence measurements were performed with a Cary Eclipse spectrofluorimeter (Varian, Mulgrave, Victoria, Australia). PBS solution of fluorescent dye ATTO-425, whose fluorescence and absorption spectra are similar to that of ThT, was taken as a reference for determining the corrected and normalized values of fluorescence intensity of ThT bound to amyloid fibrils. The fluorescence quantum yield of ATTO-425 is 0.9 (ATTO-TEC Catalogue 2009/2010 p.14). Fluorescence spectra of ThT and ATTO-425 were excited at 435 nm. The spectral slits’ width was 5 nm in all experiments. A change of the spectral slits’ width did not influence the experimental results. The registered values of fluorescence intensity were corrected on the primary inner filter effect, as described earlier [42].

### 3.9. Time-Resolved Fluorescence Measurements

The fluorescence decay curves in the subnanosecond and nanosecond ranges were recorded by a spectrometer FluoTime 300 (Pico Quant, Berlin, Germany) with the Laser Diode Head LDH-C-440 (*λ_ex_* = 440 nm). The measured emission decays were fit to a multiexponential function using the standard convolute-and-compare nonlinear least-squares procedure [61]. In this method, the convolution of the model exponential function with the instrument response function (IRF) was compared to the experimental data until a satisfactory fit was obtained. The IRF was measured using cross correlation of the excitation and fundamental gate pulse. The fitting routine was based on the nonlinear least-squares method. Minimization was performed according to Marquardt [62].

Fluorescence anisotropy was determined as: $r=\left(F_{V}^{V}-GF_{H}^{V}\right)/\left(F_{V}^{V}+2GF_{H}^{V}\right)$, where $F_{V}^{V}$ and $F_{H}^{V}$ are vertical and horizontal components of fluorescence intensity excited by vertical polarized light, and $G=F_{V}^{H}/F_{H}^{H}$ is the coefficient which determines the different sensitivity of the registering system for vertical and horizontal components of fluorescence intensity.

### 3.10. CD Spectroscopy

CD spectra in the far UV-region were measured using a J-810 spectropolarimeter (Jasco, Tokyo, Japan). Spectra were recorded in a 0.1 cm cell from 250 to 190 nm. For all spectra, an average of three scans was obtained. The CD spectrum of the appropriate buffer was recorded and subtracted from the protein spectra.

## 4. Conclusions

ThT-α-synuclein binding constants have been determined earlier in other works. For example, in the work by Ye [38] the existence of one ThT-α-synuclein binding mode with *K*_d_ value equal to 588 nM (*K*_b_ = 1.7 × 10^6^ M^−1^) was shown. Thus, the value of the binding constant obtained in the article by Ye [38] has the same order of magnitude as the binding constant, which was determined in the present work for the second mode of dye-fibril binding. However, the absolute values of these parameters are significantly different. These differences can be caused by the fact that, in most papers focused on to this problem, for dissociation constant determination the dependence of the dye fluorescence intensity (without correction of on the primary inner filter effect) on its total concentration (instead of the free ThT concentration) was used. However, this approach is incorrect and contradicts the physical meaning of the current task (see Supplementary Materials).

In this work, a novel approach based on the preparation of the tested solutions by equilibrium microdialysis for the study of the ThT-α-synuclein amyloid fibrils interaction was applied. Using this method allowed us to identify two types (modes) of dye binding to α-synucelin fibrils, with significantly different binding parameters. A greater sensitivity of the absorption spectroscopy to the determination of ThT binding parameters to the mode with a lower affinity and fluorescence spectroscopy for the mode with a higher affinity was shown. A very small number of dye binding sites for the mode with a higher affinity (1 ThT/2500 α-synuclein molecules) compared with the mode with a lower affinity (1 ThT/8 α-synuclein molecules) was shown. It should be noted that the information about ThT-α-synuclein amyloid fibrils stoichiometry can be used for estimating the correct dye/fibrils ratio in experiments on detection of this protein fibrillation.

The use of the proposed approach for tested solution preparation also allowed us to indicate significant differences in the photophysical characteristics of the dye bound to the sites of different binding modes. Analysis of the obtained results led to the suggestion that the existence of one of these modes (with lower affinity) is caused by the incorporation of the dye into the grooves formed by the side chains of amino acids along the long axis of the fibril, perpendicular to the β-sheets. Comparison of the ThT binding parameters to amyloid fibrils formed from different proteins and analysis of their images obtained by electron microscopy allowed us to assume that the existence of another mode can be caused by the localization of the dye molecules in the clustered areas of the fibrils (possibly within the above-discussed grooves along the fiber axis). The present hypothesis suggests low accessibility of this mode’s binding sites, and rigid restriction of rotation of the dye fragments relative to each other. This is in agreement with the data of a high ThT-fibrils binding affinity and low stoichiometry in this mode, as well as a high fluorescence quantum yield and lifetime of the bound dye. This assumption is also confirmed by the results of confocal microscopy of α–synuclein amyloid fibrils in the presence of ThT.

The results of this work, which made it possible to show for the first time the presence of two modes of ThT binding to α–synuclein amyloid fibrils and to characterize each of them, are currently relevant since fluorescent probes, including ThT, its analogues and its derivatives, are considered diagnostic and therapeutic agents. In experiments in vitro special attention should be given to the exact characterization of the second ThT-amyloid fibrils binding type (mode), because in the brain of patients with Parkinson’s disease α–synuclein amyloid fibrils mostly do not represent individual fibers but are a part of the pathological plaques (Lewy bodies). The formation of clusters can be a factor determining the amyloid fibrils’ cytotoxicity, or the mechanism of their effect on the cells. Therefore, in the case of such pathologies, the interaction of fluorescent probes with clusters and aggregates of amyloid fibrils can dominate over the interaction with individual fine filaments. In this regard, the results of this work may be in demand when developing approaches to determine the number of binding types of fluorescent probes to amyloid fibrils, characterize these modes, and estimate which of them is predominant in various pathologies.

## Figures and Tables

**Table 1:** Characteristics of thioflavin T (ThT) bound to amyloid fibrils and free dye in a water solution.

Conditions	*λ_abs_max_*, nm	Mode	*ε_i,_*_max_ × 10^−4^, M^−1^cm^−1^	*K_bi_* × 10^−5^, M^−1^	*n_i_*	*q_i_*
α-synuclein fibrils (by absorption spectroscopy)	438 ± 4	1	1.8 ± 0.3	0.3 ± 0.3	0.13 ± 0.20	0.02 ± 0.01
α-synuclein fibrils (by absorption and fluorescence spectroscopy)	438 ± 4	1	1.8 ± 0.3	0.3 ± 0.3	0.13 ± 0.20	0.02 ± 0.01
		2	7.0 ± 2.0	70 ± 10	(4 ± 10) × 10^−4^	0.13 ± 0.04
Insulin fibrils [41]	450	1	2.3	0.4	0.14	0.27
		2	7.9	78	0.02	0.72
Lysozyme fibrils [41]	449	1	6.2	0.6	0.25	0.0001
		2	5.3	72	0.11	0.44
Aβ42 fibrils [41]	440	1	1.4	0.2	0.26	0.03
		2	8.7	70	0.004	0.18
Free in aqueous solution [51]	412	-	3.2	-	-	0.0001

## Supplementary Materials

The following are available online at http://www.mdpi.com/1422-0067/19/9/2486/s1.

## References

[1] Ueda K., Fukushima H., Masliah E., Xia Y., Iwai A., Yoshimoto M., Otero D.A., Kondo J., Ihara Y., Saitoh T. (1993). Molecular cloning of cDNA encoding an unrecognized component of amyloid in Alzheimer disease. Proc. Natl. Acad. Sci. USA.

[2] Jakes R., Spillantini M.G., Goedert M. (1994). Identification of two distinct synucleins from human brain. FEBS Lett..

[3] Lavedan C. (1998). The synuclein family. Genome Res..

[4] Duda J.E., Shah U., Arnold S.E., Lee V.M., Trojanowski J.Q. (1999). The expression of alpha-, beta-, and gamma-synucleins in olfactory mucosa from patients with and without neurodegenerative diseases. Exp. Neurol..

[5] Nakai M., Fujita M., Waragai M., Sugama S., Wei J., Akatsu H., Ohtaka-Maruyama C., Okado H., Hashimoto M. (2007). Expression of alpha-synuclein, a presynaptic protein implicated in Parkinson’s disease, in erythropoietic lineage. Biochem. Biophys. Res. Commun..

[6] Barbour R., Kling K., Anderson J.P., Banducci K., Cole T., Diep L., Fox M., Goldstein J.M., Soriano F., Seubert P. (2008). Red blood cells are the major source of alpha-synuclein in blood. Neuro-Degener. Dis..

[7] Li Q.X., Campbell B.C., McLean C.A., Thyagarajan D., Gai W.P., Kapsa R.M., Beyreuther K., Masters C.L., Culvenor J.G. (2002). Platelet alpha- and gamma-synucleins in Parkinson’s disease and normal control subjects. J. Alzheimer’s Dis..

[8] Michell A.W., Luheshi L.M., Barker R.A. (2005). Skin and platelet alpha-synuclein as peripheral biomarkers of Parkinson’s disease. Neurosci. Lett..

[9] Noori-Daloii M.R., Kheirollahi M., Mahbod P., Mohammadi F., Astaneh A.N., Zarindast M.R., Azimi C., Mohammadi M.R. (2010). Alpha- and beta-synucleins mRNA expression in lymphocytes of schizophrenia patients. Genet. Test. Mol. Biomark..

[10] Alim M.A., Hossain M.S., Arima K., Takeda K., Izumiyama Y., Nakamura M., Kaji H., Shinoda T., Hisanaga S., Ueda K. (2002). Tubulin seeds alpha-synuclein fibril formation. J. Biol. Chem..

[11] Zhu M., Qin Z.J., Hu D., Munishkina L.A., Fink A.L. (2006). Alpha-synuclein can function as an antioxidant preventing oxidation of unsaturated lipid in vesicles. Biochemistry.

[12] George J.M., Jin H., Woods W.S., Clayton D.F. (1995). Characterization of a novel protein regulated during the critical period for song learning in the zebra finch. Neuron.

[13] Bonini N.M., Giasson B.I. (2005). Snaring the function of alpha-synuclein. Cell.

[14] Cooper A.J., Jeitner T.M., Blass J.P. (2002). The role of transglutaminases in neurodegenerative diseases: Overview. Neurochem. Int..

[15] Breydo L., Wu J.W., Uversky V.N. (2012). Alpha-synuclein misfolding and Parkinson’s disease. Biochim. Biophys. Acta.

[16] Ekman D., Light S., Bjorklund A.K., Elofsson A. (2006). What properties characterize the hub proteins of the protein-protein interaction network of Saccharomyces cerevisiae?. Genome Biol..

[17] Weinreb P.H., Zhen W., Poon A.W., Conway K.A., Lansbury P.T. (1996). NACP, a protein implicated in Alzheimer’s disease and learning, is natively unfolded. Biochemistry.

[18] Cote Y., Delarue P., Scheraga H.A., Senet P., Maisuradze G.G. (2018). From a Highly Disordered to a Metastable State: Uncovering Insights of alpha-Synuclein. ACS Chem. Neurosci..

[19] Fakhree M.A.A., Nolten I.S., Blum C., Claessens M. (2018). Different Conformational Subensembles of the Intrinsically Disordered Protein alpha-Synuclein in Cells. J. Phys. Chem. Lett..

[20] Ferreon A.C., Gambin Y., Lemke E.A., Deniz A.A. (2009). Interplay of alpha-synuclein binding and conformational switching probed by single-molecule fluorescence. Proc. Natl. Acad. Sci. USA.

[21] Veldhuis G., Segers-Nolten I., Ferlemann E., Subramaniam V. (2009). Single-molecule FRET reveals structural heterogeneity of SDS-bound alpha-synuclein. Chembiochem Eur. J. Chem. Biol..

[22] Nuscher B., Kamp F., Mehnert T., Odoy S., Haass C., Kahle P.J., Beyer K. (2004). Alpha-synuclein has a high affinity for packing defects in a bilayer membrane: A thermodynamics study. J. Biol. Chem..

[23] Pfefferkorn C.M., Jiang Z., Lee J.C. (2012). Biophysics of alpha-synuclein membrane interactions. Biochim. Biophys. Acta.

[24] Wang W., Perovic I., Chittuluru J., Kaganovich A., Nguyen L.T., Liao J., Auclair J.R., Johnson D., Landeru A., Simorellis A.K. (2011). A soluble alpha-synuclein construct forms a dynamic tetramer. Proc. Natl. Acad. Sci. USA.

[25] Lassen L.B., Reimer L., Ferreira N., Betzer C., Jensen P.H. (2016). Protein Partners of alpha-Synuclein in Health and Disease. Brain Pathol..

[26] Paleologou K.E., El-Agnaf O.M. (2012). alpha-Synuclein aggregation and modulating factors. Sub-Cell. Biochem..

[27] Wood S.J., Wypych J., Steavenson S., Louis J.C., Citron M., Biere A.L. (1999). alpha-synuclein fibrillogenesis is nucleation-dependent. Implications for the pathogenesis of Parkinson’s disease. J. Biol. Chem..

[28] Iljina M., Garcia G.A., Horrocks M.H., Tosatto L., Choi M.L., Ganzinger K.A., Abramov A.Y., Gandhi S., Wood N.W., Cremades N. (2016). Kinetic model of the aggregation of alpha-synuclein provides insights into prion-like spreading. Proc. Natl. Acad. Sci. USA.

[29] Dearborn A.D., Wall J.S., Cheng N., Heymann J.B., Kajava A.V., Varkey J., Langen R., Steven A.C. (2016). alpha-Synuclein Amyloid Fibrils with Two Entwined, Asymmetrically Associated Protofibrils. J. Biol. Chem..

[30] Zhang H., Griggs A., Rochet J.C., Stanciu L.A. (2013). In vitro study of alpha-synuclein protofibrils by cryo-EM suggests a Cu(^2+^)-dependent aggregation pathway. Biophys. J..

[31] Naiki H., Higuchi K., Hosokawa M., Takeda T. (1989). Fluorometric determination of amyloid fibrils in vitro using the fluorescent dye, thioflavin T1. Anal. Biochem..

[32] LeVine H. (1993). Thioflavine T interaction with synthetic Alzheimer’s disease beta-amyloid peptides: Detection of amyloid aggregation in solution. Protein Sci..

[33] LeVine H. (1999). Quantification of beta-sheet amyloid fibril structures with thioflavin T. Methods Enzymol..

[34] Wu C., Biancalana M., Koide S., Shea J.E. (2009). Binding Modes of Thioflavin-T to the Single-Layer beta-Sheet of the Peptide Self-Assembly Mimics. J. Mol. Biol..

[35] Biancalana M., Makabe K., Koide A., Koide S. (2009). Molecular mechanism of thioflavin-T binding to the surface of beta-rich peptide self-assemblies. J. Mol. Biol..

[36] Biancalana M., Koide S. (2010). Molecular mechanism of Thioflavin-T binding to amyloid fibrils. Biochim. Biophys. Acta.

[37] Morimoto K., Kawabata K., Kunii S., Hamano K., Saito T., Tonomura B. (2009). Characterization of type I collagen fibril formation using thioflavin T fluorescent dye. J. Biochem..

[38] Ye L., Velasco A., Fraser G., Beach T.G., Sue L., Osredkar T., Libri V., Spillantini M.G., Goedert M., Lockhart A. (2008). In vitro high affinity alpha-synuclein binding sites for the amyloid imaging agent PIB are not matched by binding to Lewy bodies in postmortem human brain. J. Neurochem..

[39] Groenning M., Norrman M., Flink J.M., van de Weert M., Bukrinsky J.T., Schluckebier G., Frokjaer S. (2007). Binding mode of Thioflavin T in insulin amyloid fibrils. J. Struct. Biol..

[40] Groenning M. (2009). Binding mode of Thioflavin T and other molecular probes in the context of amyloid fibrils-current status. J. Chem. Biol..

[41] Kuznetsova I.M., Sulatskaya A.I., Uversky V.N., Turoverov K.K. (2012). A new trend in the experimental methodology for the analysis of the thioflavin T binding to amyloid fibrils. Mol. Neurobiol..

[42] Fonin A.V., Sulatskaya A.I., Kuznetsova I.M., Turoverov K.K. (2014). Fluorescence of dyes in solutions with high absorbance. Inner filter effect correction. PLoS ONE.

[43] Sulatskaya A.I., Kuznetsova I.M., Belousov M.V., Bondarev S.A., Zhouravleva G.A., Turoverov K.K. (2016). Stoichiometry and Affinity of Thioflavin T Binding to Sup35p Amyloid Fibrils. PLoS ONE.

[44] Sulatskaya A.I., Povarova O.I., Kuznetsova I.M., Uversky V.N., Turoverov K.K. (2012). Binding stoichiometry and affinity of fluorescent dyes to proteins in different structural states. Methods Mol. Biol..

[45] Uversky V.N., Li J., Fink A.L. (2001). Evidence for a partially folded intermediate in alpha-synuclein fibril formation. J. Biol. Chem..

[46] Kjaergaard M., Norholm A.B., Hendus-Altenburger R., Pedersen S.F., Poulsen F.M., Kragelund B.B. (2010). Temperature-dependent structural changes in intrinsically disordered proteins: Formation of alpha-helices or loss of polyproline II?. Prot. Sci. Publ. Prot. Soc..

[47] Greenfield N.J. (2006). Using circular dichroism spectra to estimate protein secondary structure. Nat. Protoc..

[48] Ban T., Hamada D., Hasegawa K., Naiki H., Goto Y. (2003). Direct observation of amyloid fibril growth monitored by thioflavin T fluorescence. J. Biol. Chem..

[49] Selivanova O.M., Glyakina A.V., Gorbunova E.Y., Mustaeva L.G., Suvorina M.Y., Grigorashvili E.I., Nikulin A.D., Dovidchenko N.V., Rekstina V.V., Kalebina T.S. (2016). Structural model of amyloid fibrils for amyloidogenic peptide from Bgl2p-glucantransferase of S. cerevisiae cell wall and its modifying analog. New morphology of amyloid fibrils. Biochim. Biophys. Acta.

[50] Zahn R. (1999). Prion propagation and molecular chaperones. Q. Rev. Biophys..

[51] Sulatskaya A.I., Maskevich A.A., Kuznetsova I.M., Uversky V.N., Turoverov K.K. (2010). Fluorescence quantum yield of thioflavin T in rigid isotropic solution and incorporated into the amyloid fibrils. PLoS ONE.

[52] Oravcova J., Bohs B., Lindner W. (1996). Drug-protein binding sites. New trends in analytical and experimental methodology. J. Chromatogr. B Biomed. Appl..

[53] Krebs M.R., Bromley E.H., Donald A.M. (2005). The binding of thioflavin-T to amyloid fibrils: Localisation and implications. J. Struct. Biol..

[54] Maskevich A.A., Stsiapura V.I., Kuzmitsky V.A., Kuznetsova I.M., Povarova O.I., Uversky V.N., Turoverov K.K. (2007). Spectral properties of thioflavin T in solvents with different dielectric properties and in a fibril-incorporated form. J. Proteome Res..

[55] Sulatskaya A.I., Lavysh A.V., Maskevich A.A., Kuznetsova I.M., Turoverov K.K. (2017). Thioflavin T fluoresces as excimer in highly concentrated aqueous solutions and as monomer being incorporated in amyloid fibrils. Sci. Rep..

[56] Kuznetsova I.M., Sulatskaya A.I., Maskevich A.A., Uversky V.N., Turoverov K.K. (2016). High Fluorescence Anisotropy of Thioflavin T in Aqueous Solution Resulting from Its Molecular Rotor Nature. Anal. Chem..

[57] Sulatskaya A.I., Rodina N.P., Polyakov D.S., Kuznatsova I.M., Turoverov K.K. Investigation of amyloid fibrils on the basis of full-length and truncated forms of beta-2-microglobulin with the use of equilibrium microdialysis. Proceedings of the 7th European Conference on Biology and Medical Sciences.

[58] Sulatskaya A.I., Rodina N.P., Kuznetsova I.M., Turoverov K.K. (2017). Different conditions of fibrillogenesis cause polymorphysm of lysozyme amyloid fibrils. J. Mol. Struct..

[59] Grabenauer M., Bernstein S.L., Lee J.C., Wyttenbach T., Dupuis N.F., Gray H.B., Winkler J.R., Bowers M.T. (2008). Spermine binding to Parkinson’s protein alpha-synuclein and its disease-related A30P and A53T mutants. J. Phys. Chem. B.

[60] Vladimirov Y.A., Litvin F.F. (1964). Photobiology and spectroscopic methods. Handbook of General Biophisics.

[61] O’Connor D.V.P.D. (1984). Time-Correlated Single Photon Counting.

[62] Marquardt D.W. (1963). An algorithm for least-squares estimation of non linear parameters. J. Soc. Ind. Appl. Math..

