# Supplementary material for: Investigation of α-Synuclein Amyloid Fibrils Using the Fluorescent Probe Thioflavin T
Source: Int J Mol Sci. 2018 Aug 23;19(9):2486. doi: 10.3390/ijms19092486 (PMC6163839; doi:10.3390/ijms19092486)
Supplement: Supplementary file 1 [file ijms-19-02486-s001.pdf]

Supplementary material

Investigation of α-Synuclein Amyloid Fibrils Using the Fluorescent Probe Thioflavin T

Anna I. Sulatskaya[1], Natalia P. Rodina[1], Maksim I. Sulatsky[1], Olga I. Povarova[1], Iuliia A. Antifeeva[1], Irina M. Kuznetsova[1], Konstantin K. Turoverov[1,2,*]

[1] Laboratory of Structural dynamics, stability and folding of proteins, Institute of Cytology of the Russian Academy of Science, St. Petersburg, Tikhoretsky ave. 4, 194064, Russia

[2] Institute of Physics, Nanotechnology and Telecommunications, Peter the Great St.-Petersburg Polytechnic University, St. Petersburg, Polytechnicheskaya 29, 195251, Russia

[*]Corresponding author: K.K. Turoverov
e-mail: kkt@incras.ru
Tel.: +7 812 297 19 57
Fax: +7 812 297 35 41

1. Pitfalls of the total ThT concentration (C_0) using instead concentration of free dye (C_f) for determination of the dye binding parameters to amyloid fibrils

ThT – α-synuclein binding constants actually were determined earlier. For example, in the work [1] it was shown the existence of the one ThT – α-synuclein bindihg mode with K_d value equal to 588 nM (K_b = 1.7*10^6 M^{-1}). Thus, the value of the binding constant obtained in this work has the same order of magnitude as the binding constant, which we determined for the second mode of dye-fibril binding. However the absolute values of these parameters are significantly different. It should be noted that in work [1], as in most papers aimed to this problem, for dissociation constant determination the dependence of the dye fluorescence intensity on its total concentration was used. In order to show the incorrectness of such approach application, it is necessary to write an equation for determining the binding constant. If all the ThT binding sites in amyloid fibril are identical and independent from each other, then the binding constant of the dye to amyloid fibril (K_b) is determined as:

$$K_b = \frac{C_b}{(nC_p - C_b)\cdot C_f}.$$

where C_f is the concentrations of free ligand, C_b is the concentration of the dye bound to fibrils, i.e. the concentration of occupied binding sites, C_p is the concentration of the protein that forms fibril, n is the number of sites of ThT binding to amyloid fibril per protein molecule. Thus nC_p is the total concentration of the binding sites and (nC_p - C_b) is the concentration of free binding sites.

This means that the dependence of the bound dye concentration on the free dye concentration in solution follows a saturation curve:

$$C_b = \frac{nC_pC_f}{K_d + C_f}.$$

This equation can be rewriten as (Klots plot):

$$\frac{1}{C_b} = \frac{1}{nC_p} + \frac{K_d}{nC_p}\frac{1}{C_f}$$

Thus, for the "direct" determination of the dissociation constant, the values of C_b and C_f must be calculated. However, the determination of these values is a nontrivial task, since a solution of ThT with fibrils is always an equilibrium system of free and bound dye molecules. In this regard, in most works aimed at ThT-amyloid fibrils binding constants determination (including, in work [1]), the following equations were used:

$$F = \frac{\mathrm{F}_{\max} X}{K_d + X},$$

$$\frac{1}{F} = \frac{1}{F_{\max}} + \frac{K_d}{F_{\max}}\frac{1}{X},$$

in which the total concentration of ThT (C_0) as the X value was used instead of the concentration of the free (non-amyloid-associated) dye. Experiments based on fluorescence intensity measurement, in principle, cannot provide information about the concentration of free dye. However the replacement of the concentration of free dye with the concentration of total dye contradicts to the physical meaning of the current task. In this paper we showed how this problem can be solved using the samples obtained by the equilibrium microdialysis.

It should be noted that in most cases of the binding parameters determination, the authors do not take into account the influence of the primary inner filter effect on the recorded fluorescence intensity values. Even experienced researchers, who do not specialize in fluorescence techniques, do not take into account the fact that a plateau of the dependence of fluorescence intensity on the concentration of a fluorescent substance could not point to the saturation of binding centers, since such a character of the dependence is its general property. Furthermore, the recorded fluorescence intensity could even decrease with an increase in the content of the fluorescent substance which is also a general property of such dependences. Thus, in order to determine the correct binding parameters values, it is necessary to correct the recorded fluorescence intensity for the primary inner filter effect. In this paper, we showed how this correction can be performed using a coefficient (W) that depends only on the total optical density of the solution.

Finally, another important problem faced by researchers using the $F(C_0)$ dependence to determine ThT-amyloid fibrils binding parameters is that this approach can be applied only for the case of the one binding type existence. And in itself, this approach does not provide an opportunity to estimate the number of binding modes. In this paper, we showed that this problem can be solved using two approaches - by determining $C_b(C_f)$ using absorption spectroscopy of solutions obtained by the equilibrium microdialysis or $F(C_f)$ using fluorescence spectroscopy of the same solutions.

2. The reasons of the nonlinearity of the dependence of the fluorescence intensity on concentration and fluorescence intensity correction for the primary inner filter effect

The nonlinearity of the concentration dependence of the fluorescence intensity is caused by the so-called primary inner filter effect. The reasons for this effect include both the attenuation of the excitation light flux on its path through an absorbing solution (Beer–Lambert law) and the difference between the area that is illuminated by the excitation light and the working area from which the fluorescence light is gathered.

It is generally accepted that for low concentration solutions (low absorbance), the fluorescence intensity is proportional to the concentration of the fluorescence substance, and primary inner filter effects are negligible; however, this assumption is not valid [2]. In reality, the total fluorescence intensity is only proportional to the absorbance (A) at one point, where $A = 0$. Even at $A = 0.1$, the deviation from linearity is 12%, and at $A = 0.3$, the deviation is 38% [2]

In an ideal case when the area illuminated by the excitation light coincides with the working area from which the fluorescence light is gathered, the recorded total fluorescence intensity $F(\lambda_{ex})$ is proportional to the fraction of the excitation light that is absorbed by the solution ($1-10^{-A_\Sigma}$). If only one substance is responsible for the absorption and fluorescence of a solution, then:

$$F\left(\lambda_{ex}\right)=k'I_0\left(\lambda_{ex}\right)\Delta\lambda_{ex}\left(1-10^{-A_\Sigma}\right)q=k\frac{\left(1-10^{-A_\Sigma}\right)}{A_\Sigma}A_{FL}q \qquad (1)$$

Here, $I_0\left(\lambda_{ex}\right)\Delta\lambda_{ex}$ is the intensity of the excitation light at λ_{ex}, k' is a proportionality factor, $\Delta\lambda_{ex}$ is the spectral width of the slits of the monochromator in the excitation pathway and $k=k'I_0\left(\lambda_{ex}\right)\Delta\lambda_{ex}$ is a normalization factor determined using a standard (a fluorescent substance with known fluorescence quantum yield) at the same experimental conditions (i.e., slits widths, photomultiplier voltage, and other factors) used in the experiment with this sample. The coefficient k is chosen in a such way that the total fluorescence intensity of the standard and the sample give physical meaning to the product of absorbance and the fluorescence quantum yield:

$$F_0\left(\lambda_{ex}\right)=F\left(\lambda_{ex}\right)/W=A_{FL}q \qquad (2)$$

Here, W is a correction factor that is determined by the total absorbance of the solution at λ_{ex}:

$$W=\frac{1-10^{-A_{FL}}}{A_{FL}}. \qquad (3)$$

If there are several components in the solution that absorb $A_{FL,i}$ and fluoresce with a fluorescence quantum yield q_i, but others only absorb the excitation light with total absorbance A_{ABS}, then:

$$F_0\left(\lambda_{ex}\right)=F\left(\lambda_{ex}\right)/W=\Sigma A_{FL,i}q_i \qquad (4)$$

where $W=\frac{1-10^{-A_\Sigma}}{A_\Sigma}$ and $A_\Sigma=\sum A_{FL,i}+A_{ABS}$.

It should be noted that in most spectrofluorimeters, the detected fluorescence intensity is not proportional to the fraction of light that is absorbed by the solution. Thus, the correction factor cannot be calculated according to Eq. 4; instead, it must be determined experimentally [3]. Moreover, because the fluorescence intensity measured by these spectrofluorimeters decreases as the absorbance of the investigated solutions increases, the fluorescence of solutions with high absorbance (A > 5.0 for most spectrofluorimeters with a cell with an optical path length of 1 cm) cannot be recorded at all.

We showed experimentally [2] that the Cary Eclipse spectrofluorimeter (Agilent Technologies, Australia) enables recordings of fluorescent solutions with very high absorbance; in contrast to all known spectrofluorimeters, this spectrofluorimeter has horizontal slits (Fig. S1), [4] that allows recording the fluorescence of solutions in a much wider range of absorbance. The unique feature of this spectrofluorimeter is that the area illuminated by the excitation light coincides with the working area from which the

fluorescence light is gathered (Fig. S1). Thus, in Cary Eclipse spectrofluorimeter, the detected fluorescence intensity is proportional to the fraction of the light absorbed by the fluorescent solution. In this connection, in the present work the correction factor *W* was calculated using only the solution absorbance according to Eq. 4 for all investigated solutions of ThT with α-synuclein amyloid fibrils prepared by equilibrium microdialysis.

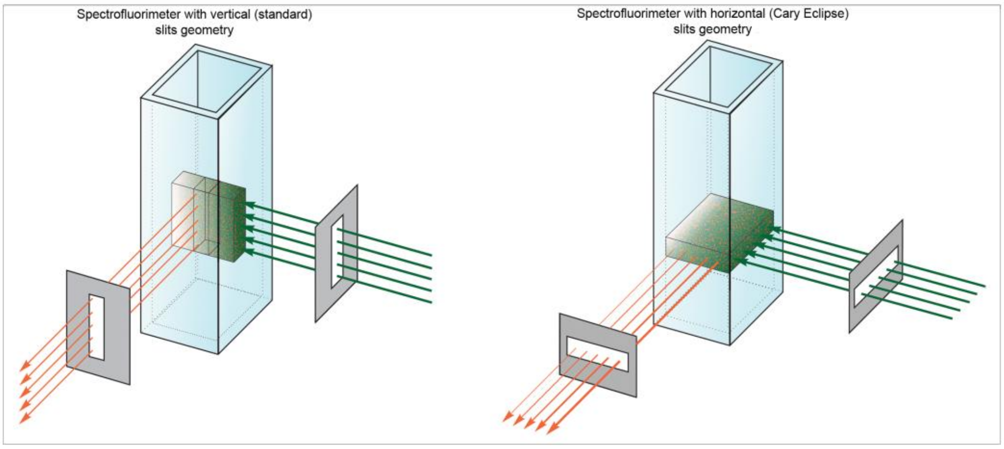

Figure S1. Schematic representation of the light flux in the spectrofluorimeters with vertical (standard) and horizontal slits geometries [2]

3. The procedure for obtaining of the spectral characteristics of ThT bound to α-synuclein amyloid fibrils by the use of equilibrium microdialysis

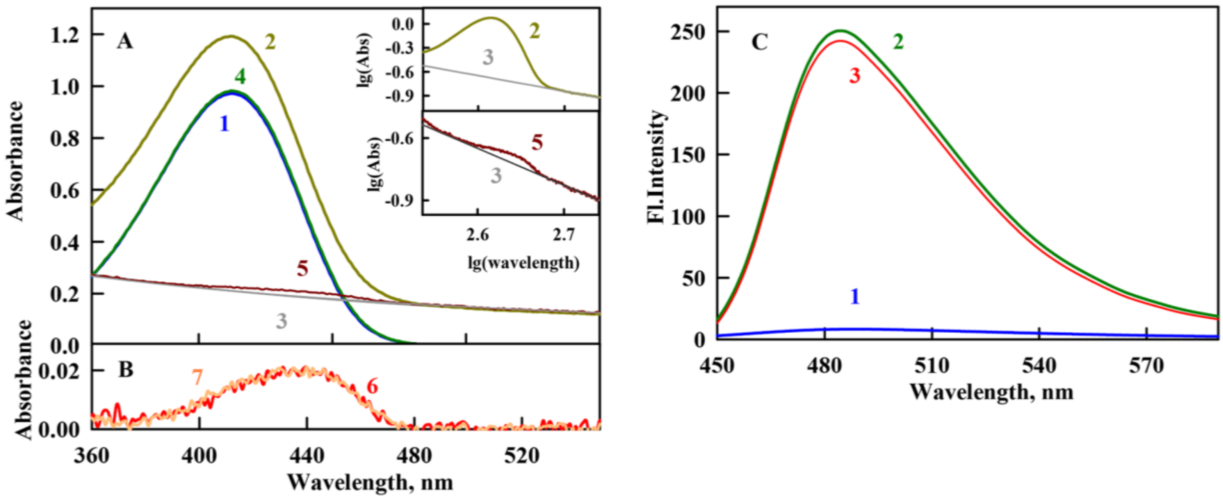

Figure S2. The use of equilibrium microdialysis for examination of ThT-α-synuclein amyloid fibrils interaction. (A) (1) - absorption spectra of ThT in chamber #2 (free ThT at concentration C_f); (2) - absorption spectra of ThT in chamber #1 (superposition of the absorption spectra of free ThT in concentration C_f, ThT bound to fibrils in concentration C_b, and the apparent absorption caused by the light scattering) after the equilibrium; (3) - optical density determined by the fibril light scattering as calculated by the equation $A_{scat}=a\lambda^{-m}$. Coefficients *a* and *m* were determined from the linear part of the curve 2 (where there is no active dye absorption) plotted in logarithmic coordinates $lg(A_{scat})=f(lg(\lambda))$ (see Insert, curve 3). (4) - total absorption of free and bound dye after light

scattering subtraction ($A(\lambda)\#2 - A_{scat}$); (5) - absorption spectrum obtained by measuring the difference spectrum between the spectra of solutions from chambers #1 and #2 (superposition of the absorption spectra of bound to fibrils ThT and the apparent absorption caused by the light scattering). (B) Absorption spectra of ThT incorporated into α-synuclein amyloid fibrils (6) - evaluated as $A_b(\lambda)=A(\lambda)\#2 - A(\lambda)_{scat} - A(\lambda)\#1$ (the difference between the spectra (4) and (1)) and (7) - obtained from spectrum (5) after light scattering subtraction (calculation procedure is shown in the Insert). (C) - (1) corrected on the primary inner filter effect fluorescence spectra of ThT in chamber #2 (free ThT); (2) - corrected on the primary inner filter effect fluorescence spectra of ThT in chamber #1 (superposition of the fluorescence spectra of free and bound to fibrils ThT); (3) - fluorescence spectrum of ThT incorporated into α-synuclein amyloid fibrils (the difference between the spectra (2) and (1)).

Absorption spectra (6) and (7) were averaged. Obtained absorption and fluorescence spectra of free and bound to fibrils ThT were smoothed and normalized to unity at the spectra maximum and presented at the Figure 3.

4. Statistical analysis of the experimental data approximation by the models of one and two modes (types) of ThT binding to α-synuclein amyloid fibrils

A statistical analysis of the results obtained under the assumption of existence of one and two binding modes was performed.

	Ā, %	ρ	F-statistics	R^2	d
1 binding mode	36	0.72	50.1	0.85	0.5
2 binding modes	14	0.95	52.2	0.89	2.1

We showed that the approximation under the assumption of two bonding modes has better values of the model characteristics compared with approximation under the assumption of one binding mode: a smaller average error of approximation Ā (which indicates better mathematical accuracy); a larger correlation index ϱ (which indicates a stronger nonlinear coupling); a larger calculated value of the Fisher's F-test (which indicates a more adequate description of the experimental data and the statistical significance of the approximation); a larger adjusted coefficient of determination R2 (which indicates a better agreement between the experimental data and the approximating curve). A comparison of the models residuals was made and it was shown that the residuals of the two binding modes model are much smaller than the residuals of the model of one binding mode ($P < 5 \cdot 10^{-5}$). The models bias (t-test) was estimated and it was shown that the model of one binding mode is biased, and the model of two binding modes is unbiased. In addition, using the Durbin-Watson statistics (d) for a model of one binding mode (in contrast to the model of two binding modes) positive residuals autocorrelation was shown, which indicates the unsatisfactory nature of this approximation. Thus, using careful statistical analysis the better fit by the two-site binding model approximation was proved.

References

Ye, L., Velasco, A., Fraser, G., Beach, T. G., Sue, L., Osredkar, T., Libri, V., Spillantini, M. G., Goedert, M., Lockhart, A. (2008) In vitro high affinity alpha-synuclein binding sites for the amyloid imaging agent PIB are not matched by binding to Lewy bodies in postmortem human brain. J. Neurochem. 105, (4), 1428-37.

Fonin, A. V., Sulatskaya, A. I., Kuznetsova, I. M., and Turoverov, K. K. (2014) Fluorescence of dyes in solutions with high absorbance. Inner filter effect correction. PloS one 9, e103878.

Haidekker, M. A., and Theodorakis, E. A. (2010) Environment-sensitive behavior of fluorescent molecular rotors. J. Biol. Eng. 4, 11.

Steensrud, J. (2012) Selecting the correct spectrophotometer for quality results. Agilent Technologies, 25.
